# Practical Issues in Managing Systemic Inflammatory Disorders During the COVID-19 Pandemic

**DOI:** 10.31138/mjr.31.3.253

**Published:** 2020-09-08

**Authors:** Theodoros Dimitroulas, George Bertsias

**Affiliations:** 1 4^th^ Department of Internal Medicine, Hipokration Hospital, School of Medicine, Aristotle University of Thessaloniki, Thessaloniki, Greece; 2 Rheumatology and Clinical Immunology, University of Crete Medical School and University Hospital of Iraklio, Iraklio, Greece

**Keywords:** COVID-19, systemic diseases, drug monitoring, rheumatoid arthritis, systemic lupus erythematosus

## Abstract

The global coronavirus disease 2019 (COVID-19) situation threatens not only the health of populations, but also the coherence and function of health care systems. Patients with systemic inflammatory disorders feel the overwhelming strain of COVID-19, since their disease, administered treatments, and associated comorbidities may all contribute to increased vulnerability to infection. At the same time, monitoring the activity status of rheumatic diseases and adjusting the treatments where appropriate, are important for preventing flares and other complications, which could pose additional health risks. Considering the urgent need to maintain physical distancing and self-quarantine as much as possible, we herein discuss the challenges and possible solutions pertaining to the assessment and monitoring of patients with systemic inflammatory diseases. We also discuss issues related to the prescription and supply of anti-rheumatic drugs, as well as opportunities provided by the use of technological and wireless tools. From an optimistic viewpoint, the end of this pandemic may leave us with an important legacy in utilising and implementing e-health solutions that may both improve the clinical care standards for patients with systemic inflammatory diseases and also reduce the burden placed on healthcare systems.

## INTRODUCTION

The coronavirus disease 2019 (COVID-19) pandemic has posed unprecedented challenges on physicians, patients, and health systems with several unanswered questions regarding the course, treatment, and long-term outcomes of the disease. The rapid and uncontrolled dissemination of the infection globally has clearly raised concerns about the care of several groups of patients, especially immunocompromised individuals such as those suffering from cancer, haematological malignancies, systemic inflammatory diseases, as well as transplanted persons. Particularly, in the case of rheumatic patients, the immune response to virus is perceived to be lower, due to both the disease itself and iatrogenic effects associated with the administration of corticosteroids and immunosuppressive drugs. On the other hand, maintaining disease remission in patients with systemic inflammatory disorders during a pandemic infection is of outmost importance for several reasons including the higher risk of infection in sub-optimally controlled disease state and flare-ups,^1^ as well as the avoidance of unnecessary office visits, emergency rooms attendances and inpatient admissions all of which exacerbate the danger of exposure to COVID-19.^2,3^ To this end, International Rheumatology Societies advise against the discontinuation of immunosuppressive medications in asymptomatic patients or those who have not been in contact with infected subjects.^4,5^ Nonetheless, an individualized approach based on the exposure risk to the virus, the type and the severity of the underlying disease, the way of administration of immunomodulatory treatment (oral, subcutaneous, intravenous) coupled with the presence of emerging risk factors for severe COVID-19 disease, namely older age, male sex, hypertension, obesity, cardiovascular or respiratory comorbidities and diabetes mellitus,^6^ is strongly recommended to minimize the risks of infection and adverse outcome.^7^ As this is a rapidly evolving situation and robust data for the true impact of COVID-19 on patients with autoimmune diseases is lacking, it is likely that such recommendations will change based on emerging evidence and the infection burden.^8^ In this context of growing health emergency and depending on local conditions such as the rate of community and/or country virus spread, availability of medical staff and movement restrictions imposed by governors, routine, non-urgent appointments in stable patients could be postponed or delayed. However, under these circumstances, the regular follow-up of disease activity, the monitoring of anti-rheumatic drug’s effectiveness and toxicity with the relevant laboratory tests, the continuity in medicines’ access, and, more importantly, the surveillance of rheumatic patients regarding COVID-19 infection all become very challenging and highly demanding tasks. Rheumatology departments have to adapt to this new situation and increase their flexibility to provide advice and care to patients with systemic rheumatic diseases.

## RHEUMATOLOGY OUTPATIENTS’ VISITS AND MONITORING

Even during the COVID-19 outbreak, patients with systemic rheumatic disorders need to continue to have some kind of follow-up to monitor disease status and safe administration of treatment. For clinically quiescent patients, visits could be rescheduled or transitioned to telemedicine in order to minimize physical contact for both medical professional and patients. Clinic staff should provide information about preferred telephone or video platforms for online consultations and documentation (x-rays, MRI scans review, etc). Of note, a number of patient-completed indices exist have been validated for assessment of disease activity status in rheumatoid arthritis (eg, RAPID3),^9^ ankylosing spondylitis (eg, BASAI, BASFI) and lupus (Systemic Lupus Activity Questionnaire [SLAQ]),^10^ and thus may be useful tools, especially for patients who have already been acquainted in using them. However, major therapeutic decisions such as initiation, switching or discontinuation of DMARDs should be based on objective clinical assessment coupled with the patient self-assessment of her/his own disease status. Remote monitoring for laboratory investigations could be implemented by electronic messages sent directly by patients and/or laboratories to departmental portals or to physicians’ e-mail address. Should routine blood tests be safely delayed after clinical judgment including consideration of dose and duration of treatment, prior haematological or biochemical abnormalities as well as accommodations for social distancing at laboratory, patients should be advised accordingly with accurate guidance when and where to get blood work done.

In all instances, patients should be contacted by telephone for any new or concerning symptoms, as face-to-face visits may be inevitable in many occasions, such as new cases with evolving symptoms, or patients at risk for significant negative outcomes without evaluation. In this case use of appropriate personal protective equipment (masks, gloves, etc.) and appointments when volumes are lowest are essential. Last but not least, patients should be encouraged to call their physicians with questions, especially medical problems, between visits, providing that such communications are in line with national Data protection regulations.

## ASSESSMENT OF RHEUMATIC PATIENTS FOR COVID-19 DISEASE

Traditionally, patients with systemic autoimmune diseases are classified as being at higher risk for infections. Nonetheless, the “net” state of immunosuppression may show inter-individual variation and also depend on the dose and immunosuppressive potency of administered treatments.^11^ Accordingly, such individuals should be particularly stringent in following recommendations for social distancing measures and unnecessary movement. In case of appearance of symptoms suggestive of COVID-19 infection such as fever, cough and dyspnoea, the primary care provider or rheumatologist are probably the first to be contacted via telephone or other remote device. Although self-isolation is recommended if symptoms are mild, the immunosuppressive net-state of rheumatic patients imposes more urgent action regarding testing for COVID-19 compared to the general population accompanied by the necessity for prompt clinical assessment especially if symptoms persist. Immediate rheumatology consultation regarding anti-rheumatic therapy and consideration for impatient care should be considered after individualized approach.^12^

## DRUG PRESCRIPTION FOR RHEUMATIC PATIENTS DURING THE PANDEMIC

The COVID-19 pandemic has also imposed significant challenges on the prescription and access to chronic disease treatments, including anti-rheumatic drugs, due to multiple reasons such as the infrequency or even cancelling of patient visits, movement restrictions and possible shortages in the production and/or supply of selected drug compounds. To overcome these, several countries across the globe have enabled fast-track electronic prescription processes to help protect people most at-risk in the community from possible exposure to the virus.^13,14^ Through special arrangements with the pharmacists, drug prescriptions (including biologics) can now be dispensed in “paperless” ways, such as via barcoded e-mails or text messages sent to smartphones, without the need for wet signatures. Similarly, dispensing of synthetic and biologic DMARDs can be automatically renewed up to a period that varies across countries from three to twelve months, which also helps to minimize visits to physicians and pharmacists. Such practice, however, should be accompanied by vigilant patient monitoring as outlined above, in order to avoid any unnecessary drug exposure and associated adverse events.

Another relevant issue is the physical process of obtaining drug refills, including biological (or targeted synthetic) DMARDs, in particular the need for patients (or their authorized proxies) to move to pharmacies and get the needed medications. To facilitate this, some national health care systems have gradually implemented free-of-charge drug delivery options, and similar initiatives are also supported by several drug pharma companies in the context of their Patient Support Programs.

Finally, concerns have been raised over the possible shortage in the production and/or supply of certain medications including anti-rheumatic agents. Although, to date, the COVID-19 pandemic has not significantly impacted industrial drug production, the availability of certain agents has been challenged due to their redirection for the treatment of COVID-19 infection and/or its complications.^15^ A notable example is hydroxychloroquine, officially labelled for the treatment of cutaneous lupus, systemic lupus erythematosus, and rheumatoid arthritis, which has yielded signs for possible efficacy against the COVID-19 virus.^16,17^ This caused a tremendous worldwide increase in the demand for drug supply by the hospital units treating COVID-19 cases. At the same time, publicity over the possible therapeutic use of the drug led to excessive drug prescription to outpatients. Inevitably, hydroxychloroquine supplies ran into temporary shortage, thus causing anxiety to Rheumatologists and patients, especially those with SLE, due to the well-known risk for disease exacerbation upon drug withdrawal. Coordinated actions to set more stringent rules for hydroxychloroquine prescription and increase global drug production has gradually restored the supply and availability of the compound although close monitoring of the situation will be required over the ensuring months.

## COVID-19 PANDEMIC AS AN OPPORTUNITY TO IMPLEMENT E-HEALTH IN RHEUMATIC DISEASES

Over the last decade “treat-to-target” has become a standard treatment strategy leading to improved long term outcomes for patients suffering from systemic rheumatic diseases.^18^ The tight control and monitoring of disease activity as well as the adjustment of treatment to accomplish the predefined target, require frequent follow-up visits which are difficult to be achieved with the traditional management approach in rheumatology clinics. Therefore, there is an emerging need for the implementation of e-health applications in daily clinical practice, which can allow the reduction of face-to-face communication, the easier collection of clinical data, as well as the engagement of patients in treatment and other health-related decisions. Mobile health systems consisting of personal technology tools such home PCs or mobile phones, remote video consultation, virtual clinics and other web-based applications could overcome barriers of rheumatology personnel shortage, timely access to health information, and prioritisation of patients who may require a clinical evaluation and prevent delay in treatment modification.^19^ However, remote patient monitoring in the field of rheumatic diseases is in its infancy compared to other chronic diseases, such as diabetes mellitus and hypertension, despite some promising data from large registries and clinical trials.^20,21^ Undoubtedly, mobility and other restrictions due to the COVID-19 pandemic represent an excellent opportunity not only for rheumatologists, but also for the patients to familiarise themselves with modern technology modalities and innovative tools to implement remote monitoring and consulting in the daily practice setting. Given that the duration of the post-pandemic period is uncertain, with some models predicting that epidemiologic surveillance should be maintained as late as 2024 to anticipate the possibility of COVID-19 resurgence,^22^ the appropriate use of e-health modalities may generate unlimited possibilities for various types of interaction between rheumatology care providers and patients, and result in transformation of rheumatology care by effective communication and empowerment of patients. The design of user-friendly, flexible electronic tools able to fulfil disease-specific needs, the support of adaptable wireless technologies, the minimisation of technical problems which interrupt network communication as well as the compliance with legal and ethical issues are important aspects for the successful introduction and widest usage of modern technology applications during this period and the future.

## CONCLUSION

The COVID-19 pandemic inevitably affects the care of patients with complex rheumatic diseases on several grounds, but also conveys unique challenges and opportunities for rheumatology centres. The optimal management of patients with systemic diseases remains the overhanging goal of rheumatology practice during this lockdown period despite practical issues and travel restrictions. The incorporation of modern technology tools could assist in remote monitoring and medical advice when required in order to ensure the best possible outcomes for rheumatic patients during this pandemic period.
